# Understanding Alcohol Relapse in Liver Transplant Patients With Alcohol-Related Liver Disease: A Comprehensive Review

**DOI:** 10.7759/cureus.54052

**Published:** 2024-02-12

**Authors:** Shreyashee S Shinde, Swarupa Chakole, Sonal Humane

**Affiliations:** 1 Pediatrics, Jawaharlal Nehru Medical College, Datta Meghe Institute of Higher Education & Research, Wardha, IND; 2 Community Medicine, Jawaharlal Nehru Medical College, Datta Meghe Institute of Higher Education & Research, Wardha, IND; 3 Mental Health Nursing, Smt. Radhikabai Meghe Memorial College of Nursing, Datta Meghe Institute of Higher Education & Research, Wardha, IND

**Keywords:** prevention strategies, multidisciplinary care, holistic approach, alcohol relapse, liver transplantation, alcohol-related liver disease

## Abstract

Alcohol-related liver disease (ALD) presents a significant global health concern, with liver transplantation being a crucial intervention for patients in the advanced stages of the disease. However, the persistent risk of alcohol relapse in transplant recipients with ALD remains a formidable challenge. This comprehensive review explores the multifaceted nature of alcohol relapse, from its underlying factors to strategies for prevention. It highlights the importance of rigorous pre-transplant assessments, effective post-transplant interventions, and the role of multidisciplinary care teams in mitigating the risk of relapse. Furthermore, the review underscores the significance of adopting a holistic approach to ALD and transplantation, acknowledging the interconnectedness of medical, psychosocial, and psychological factors. With this holistic approach, we aim to enhance patient outcomes, reduce relapse rates, and ultimately improve the overall quality of life for individuals affected by ALD.

## Introduction and background

Alcohol-related liver disease (ALD) is a chronic and progressive medical condition characterized by liver damage resulting from the long-term and excessive consumption of alcohol. ALD encompasses a spectrum of liver disorders, including fatty liver, alcoholic hepatitis, fibrosis, and cirrhosis. The impact of ALD on individuals and public health is significant, making it a global concern [1]. The pathophysiology of ALD involves various mechanisms, including the direct toxic effects of alcohol on liver cells, oxidative stress, inflammation, and the activation of fibrogenic pathways. Over time, these processes can lead to severe liver damage, ultimately necessitating medical intervention [2].

Liver transplantation is a life-saving medical procedure that plays a crucial role in the management of advanced ALD. When ALD progresses to end-stage cirrhosis and conventional treatments are no longer effective, liver transplantation offers a potential cure. It replaces the damaged liver with a healthy donor organ, enabling patients to regain a better quality of life [3]. Liver transplantation has been a remarkable advancement in ALD treatment, significantly improving many patients' survival rates and quality of life. However, it also presents a unique set of challenges, particularly concerning the risk of alcohol relapse [4].

The prevalence rate of alcohol relapse in liver transplant patients with ALD varies across studies. Reported post-transplant alcohol relapse rates in recipients with ALD range from 15% to 50% [5]. Despite the success of liver transplantation in ALD treatment, one pressing issue remains the risk of alcohol relapse in transplant recipients. Alcohol relapse, defined as the resumption of alcohol consumption following transplantation, can have severe consequences. It not only jeopardizes the survival of the transplanted organ but also poses a threat to the overall well-being of the patient [5]. This problem raises critical questions about the factors contributing to relapse, the methods for assessing and predicting the risk, and the strategies for preventing relapse in this population. Understanding the dynamics of alcohol relapse in liver transplant patients is essential to ensure the long-term success of these life-saving procedures [6].

The primary purpose of this comprehensive review is to explore and elucidate the multifaceted issue of alcohol relapse in liver transplant patients with ALD. We aim to provide a comprehensive examination of the problem by delving into its underlying causes, assessing risk factors, and evaluating current strategies for prevention. By consolidating the existing knowledge and insights into this topic, this review aspires to contribute to a more profound understanding of alcohol relapse in this particular patient population.

## Review

Alcohol-related liver disease and its impact

Definition and Types of ALD

Alcoholic fatty liver (steatosis): This is the initial and often reversible stage of ALD. It occurs when excessive alcohol consumption leads to the accumulation of fat within liver cells. This fat buildup is primarily due to the disruption of normal fat metabolism in the liver, which is a consequence of alcohol's toxic effects on the liver. Alcoholic fatty liver is usually asymptomatic, and individuals may not even be aware that they have this condition. However, it serves as an early warning sign that the liver is being harmed by alcohol. With abstinence from alcohol, this condition can often be reversed, and liver function can return to normal. If alcohol consumption continues, this condition can progress to more severe forms of ALD [7].

Alcoholic hepatitis: Alcoholic hepatitis is an acute inflammatory condition of the liver, typically resulting from prolonged heavy drinking. It is more severe than alcoholic fatty liver and is characterized by inflammation and damage to liver cells. Symptoms of alcoholic hepatitis may include jaundice (yellowing of the skin and eyes), abdominal pain in the upper right side, fever, and, in severe cases, hepatic encephalopathy, which is a condition that affects brain function due to liver dysfunction. Alcoholic hepatitis can be life-threatening and often requires immediate medical attention. Abstinence from alcohol at this stage can improve the prognosis, but some damage may be irreversible [8]. The rise in AST and ALT levels in hepatic function tests can vary depending on the underlying condition. In general, AST and ALT levels are elevated in liver diseases such as hepatitis, cirrhosis, and liver cancer. According to a study, the mean AST and ALT levels in patients with alcoholic liver disease were 98.5 U/L and 87.5 U/L, respectively [3]. Another study found a significant increase in AST levels during altitude acclimatization, indicating potential liver injury [4]. However, it is important to note that the specific rise in AST and ALT levels can vary depending on the individual and the underlying condition.

Alcoholic cirrhosis: Cirrhosis is the advanced stage of ALD, representing extensive scarring of the liver tissue. This scarring occurs due to the body's attempt to repair the ongoing liver damage caused by chronic alcohol abuse. Cirrhosis is associated with severe liver dysfunction and is considered the end-stage of ALD. Symptoms of cirrhosis can be debilitating and may include ascites (fluid buildup in the abdomen), portal hypertension (increased pressure in the blood vessels leading to the liver), and an increased risk of liver cancer. Cirrhosis is not reversible, and the liver's ability to function is significantly compromised. Liver transplantation may be the only treatment option for individuals with severe alcoholic cirrhosis who do not respond to other therapies [9].

Prevalence and Incidence of ALD

Global burden: ALD is indeed one of the leading causes of liver-related morbidity and mortality worldwide. It is a significant public health concern that substantially impacts the global population. ALD contributes significantly to the burden of liver diseases and is a leading cause of liver-related complications, including cirrhosis and liver cancer. ALD is a significant driver of global liver-related morbidity and mortality. Around the world, in 2016, alcohol use was associated with 3 million deaths (5.3% of all deaths), surpassing hypertension and diabetes combined. The global burden of ALD underscores the need for awareness, prevention, and effective management of this condition [10].

Regional disparities: The prevalence of ALD can vary significantly from one region to another, primarily due to differences in alcohol consumption patterns. Regions with higher rates of alcohol consumption tend to have a greater prevalence of ALD. However, it is important to note that ALD is not limited to any specific geographic area and can be a significant concern in many parts of the world. In regions where alcohol consumption is culturally ingrained, there may be a higher incidence of ALD. Still, it is essential to recognize that this disease affects people globally, regardless of location [11].

Rising trends: The prevalence of ALD can be influenced by changing patterns of alcohol consumption. If there is an increase in heavy or binge drinking within a population, it can lead to a rise in ALD cases. Public health authorities must monitor these trends and adapt their strategies accordingly. Education, awareness campaigns, and policies aimed at reducing harmful alcohol use can play a significant role in preventing and mitigating ALD. Additionally, healthcare systems should be prepared to manage the healthcare needs of individuals affected by ALD, as the prevalence may change over time [12].

Pathophysiology of ALD

Direct toxicity: The direct toxicity of alcohol and its metabolites, such as acetaldehyde, plays a central role in the development of ALD. This toxicity can lead to liver inflammation, steatosis, and fibrosis, ultimately progressing to more severe conditions such as cirrhosis and hepatocellular carcinoma [2-4]. Additionally, regulatory factors, transcription factors, biochemical enzymes, and miRNAs in the liver are altered in the context of ALD, contributing to its pathogenesis [13]. The pathophysiology of ALD is complex and involves a combination of environmental, genetic, and molecular factors, making it a multifaceted area of study.

Oxidative stress: Alcohol consumption is known to increase oxidative stress within the liver. Oxidative stress occurs when there is an imbalance between the production of harmful molecules called free radicals and the body's ability to neutralize them with antioxidants. Excessive alcohol intake can lead to the overproduction of free radicals, which can damage cellular structures and DNA. This oxidative damage can further contribute to liver inflammation and injury [14].

Inflammation: Chronic alcohol consumption triggers an inflammatory response in the liver. This inflammation is the body's natural defense mechanism against damage and injury. However, when this response is chronic and sustained due to ongoing alcohol consumption, it can become detrimental. Inflammatory processes can harm liver cells and lead to hepatocellular injury. This sustained inflammation is a critical factor in the progression of liver damage [15].

Fibrosis and cirrhosis: Repeated injury and inflammation in the liver can lead to the deposition of scar tissue, a process known as fibrosis. As fibrosis continues to accumulate, it can progress to cirrhosis, which is the advanced scarring of the liver tissue. Cirrhosis is characterized by extensive and irreversible damage, significant loss of liver function, and the development of complications like portal hypertension, ascites, and an increased risk of liver cancer. Cirrhosis is the end-stage of alcoholic liver disease and represents a severe and life-threatening condition [16].

Clinical Manifestations and Complications

Fatigue and weakness: Persistent fatigue and weakness are common symptoms of ALD. These symptoms can result from the liver's reduced ability to metabolize and store energy, as well as the overall impact of chronic liver damage on the body. Fatigue and weakness can significantly affect a patient's quality of life and ability to perform daily activities [17].

Jaundice: Jaundice is a well-known sign of liver dysfunction. In ALD, the liver's impaired ability to process bilirubin (a waste product from the breakdown of red blood cells) can lead to a buildup of bilirubin in the blood, resulting in the yellowing of the skin and the whites of the eyes. Jaundice is a visible indicator of liver problems [17].

Ascites: Ascites is fluid accumulation within the abdominal cavity. It occurs as a complication of advanced cirrhosis, where the liver's scarring obstructs blood flow through the organ. This increases pressure in the veins that carry blood to the liver, leading to fluid leakage into the abdomen. Ascites can cause abdominal distention, discomfort, and difficulty breathing. It is a sign of significant liver dysfunction and portal hypertension [17].

Portal hypertension: Cirrhosis, particularly advanced cirrhosis, can lead to an increase in pressure within the portal vein, a large blood vessel that carries blood from the digestive organs to the liver. This increased pressure can result in several complications, including variceal bleeding (bleeding from enlarged blood vessels in the esophagus or stomach) and splenomegaly (enlargement of the spleen). Portal hypertension is a hallmark of severe liver damage in ALD [17].

Hepatic encephalopathy: Hepatic encephalopathy is a neurological condition that can occur as a result of liver dysfunction. When the liver cannot effectively detoxify harmful substances, such as ammonia, they can accumulate in the blood and affect the brain. Patients with hepatic encephalopathy may experience confusion, disorientation, impaired cognition, and, in severe cases, coma. It is a serious complication that often requires medical intervention [17].

Liver cancer: Patients with cirrhosis, whether caused by ALD or other factors, are at an increased risk of developing hepatocellular carcinoma, a type of liver cancer. Chronic liver damage and regenerative response to injury can lead to the development of cancerous cells. Regular surveillance and early detection of liver cancer are crucial in patients with cirrhosis to improve treatment outcomes [17].

Liver transplantation in ALD

Eligibility Criteria for Liver Transplantation in ALD Patients

Severity of liver disease: Transplant eligibility is typically determined based on the severity of liver damage. The Model for End-Stage Liver Disease (MELD) score is widely used to assess the severity of liver disease and prioritize transplant candidates. Patients with advanced cirrhosis, as indicated by a high MELD score and the presence of complications like ascites, hepatic encephalopathy, and variceal bleeding, are often considered strong candidates for transplantation due to the life-threatening nature of their condition [18].

Abstinence period: Most transplant centers require patients to demonstrate a period of sustained alcohol abstinence before being considered for a liver transplant. The abstinence period serves as an essential component of the evaluation process and is designed to ensure the patient's commitment to sobriety. This period can vary but is typically around six months or longer. It provides an opportunity for the patient to prove their ability to maintain sobriety and reduce the risk of recurrent ALD post-transplant [19].

Psychosocial evaluation: A comprehensive psychosocial assessment is a critical component of the evaluation process. This evaluation assesses the patient's psychological and social readiness for transplantation. It includes examining the patient's support system, coping mechanisms, and potential for relapse. The goal is to ensure that the patient has the necessary resources and resilience to maintain abstinence and adhere to post-transplant care [20].

Medical and psychological evaluation: Patients undergo thorough medical and psychological evaluations to assess their overall health and mental well-being. These evaluations are essential to identify any comorbid conditions or psychological issues that might affect the patient's suitability for transplantation and post-transplant recovery. It also helps ensure that the patient is physically and mentally prepared for the transplant procedure and the subsequent recovery [21].

Social support: A strong social support system is often considered essential for the success of the transplant and post-transplant care. This support system typically includes family, friends, and healthcare providers who can provide emotional support and help with the patient's physical and psychological needs during the recovery process. A lack of social support can be a challenging factor in the evaluation process [22].

Outcomes and Survival Rates After Transplantation

Improved survival: Liver transplantation offers the best chance for survival in patients with end-stage ALD. When patients with advanced cirrhosis and complications of ALD receive a transplant, their post-transplant survival rates are generally favorable. The new liver can effectively perform the essential functions the diseased liver could no longer manage. However, it is important to note that the long-term success of the transplant is closely tied to the patient's commitment to remaining abstinent from alcohol. Abstinence is crucial to prevent the recurrence of ALD in the transplanted liver and to ensure a successful outcome [23].

Reduced complications: Transplantation can effectively address the complications associated with advanced ALD. Conditions like ascites (abdominal fluid accumulation), hepatic encephalopathy (neurological dysfunction due to liver disease), and variceal bleeding (bleeding from enlarged blood vessels in the esophagus or stomach) are common complications of advanced cirrhosis in ALD. A successful liver transplant can resolve these complications by providing a healthy, functioning liver that can maintain normal blood clotting, fluid balance, and metabolic functions [24].

Enhanced quality of life: Liver transplantation can significantly enhance the quality of life for ALD patients. After a successful transplant, patients often experience a remarkable improvement in their overall well-being. They can regain their health, return to work, engage in social activities, and enjoy a more typical lifestyle. The absence of the debilitating symptoms and complications associated with advanced ALD allows patients to lead a more fulfilling and active life. This is particularly meaningful for individuals who had been severely limited by the effects of their liver disease before the transplant [25].

Challenges and Limitations in ALD Liver Transplantation

Risk of alcohol relapse: Perhaps the most significant challenge in ALD liver transplantation is the risk of alcohol relapse. Despite stringent pre-transplant abstinence criteria, a portion of patients may struggle with maintaining sobriety after the transplant. Alcohol relapse can have detrimental consequences, potentially leading to recurrent ALD and failure of the transplanted liver. It requires close monitoring, support, and interventions to minimize the risk of relapse [26].

Organ shortage: The availability of donor organs is a critical concern in transplantation, not only for ALD but for all liver transplant candidates. There is a shortage of organs for transplantation, leading to long waiting lists and potential delays in receiving a transplant. This shortage underscores the need for increased organ donation and improved strategies for organ procurement and distribution to ensure timely access to transplants for patients with ALD and other liver diseases [27].

Post-transplant care: ALD transplant recipients require lifelong medical follow-up and adherence to immunosuppressive medications to prevent organ rejection. This necessitates comprehensive and coordinated post-transplant care. Patients must have access to medical professionals experienced in liver transplantation to monitor their health, manage medications, and address any potential complications [28].

Resource allocation: The allocation of donor organs, especially to patients with a history of alcohol abuse, raises ethical and resource allocation concerns. Some argue that these organs could benefit non-ALD patients, mainly when there is a shortage of available organs. This debate underscores the importance of careful and transparent organ allocation policies to ensure fairness and equity in distributing a limited resource [29].

Psychosocial support: Providing adequate psychosocial support to help ALD patients maintain sobriety after transplantation is a crucial but challenging aspect of care. Patients often require ongoing counseling, addiction treatment, and support from healthcare professionals, family, and peers to remain alcohol-free. Developing effective strategies for psychosocial support and relapse prevention is essential to enhance the long-term success of liver transplantation in ALD patients [30].

Factors contributing to alcohol relapse

Psychosocial Factors

Social support: The presence of a strong and reliable social support system is a crucial factor in maintaining sobriety after a liver transplant. Support from family, friends, and support groups can significantly impact an individual's ability to stay alcohol-free. Supportive relationships provide encouragement, understanding, and accountability, all of which can help the individual cope with the challenges of post-transplant life. Family members and close friends can play a vital role in helping the individual avoid situations that may trigger relapse [31].

Stressors: Life stressors, such as financial problems, relationship issues, or work-related stress, can be powerful triggers for relapse. Coping with and managing stress is a critical aspect of sobriety maintenance. Patients must develop effective stress management strategies, including therapy, mindfulness techniques, or stress reduction practices. Addressing the root causes of stress and finding healthier ways to cope is essential in preventing relapse [32].

Mental health: Co-occurring mental health disorders, such as depression, anxiety, or post-traumatic stress disorder, can increase the risk of alcohol relapse. Proper assessment, diagnosis, and treatment of these mental health concerns are essential. The presence of untreated mental health issues can lead individuals to self-medicate with alcohol, making it even more critical to address both alcohol addiction and co-occurring mental health disorders simultaneously. Therapy, medication, and counseling can be integral components of this treatment [33].

Biological Factors

Genetic predisposition: The role of genetics in alcohol addiction is a complex and well-documented aspect of the disease. Genetic factors can contribute to an individual's vulnerability to addiction, making some people more genetically predisposed to alcohol dependence. These genetic predispositions can involve gene variations related to alcohol metabolism, neurotransmitter function, and the brain's reward pathways. For example, specific genetic variants may influence how quickly alcohol is metabolized, affecting an individual's sensitivity to its effects and the risk of developing alcohol dependence. Understanding genetic predisposition is essential in the field of addiction medicine because it allows for the identification of those at higher risk. This knowledge can help inform prevention strategies, such as targeted education and support for individuals with a family history of addiction [34].

Neurobiology of addiction: Addiction is not merely a matter of willpower but involves profound changes in the brain's neurobiology. The neurobiological aspects of addiction are linked to alterations in the brain's reward and decision-making systems. Chronic alcohol use can disrupt the normal functioning of these brain regions, leading to persistent cravings and a reduced ability to resist the urge to drink. For instance, the brain's reward system becomes hypersensitive to alcohol's pleasurable effects, while the brain's prefrontal cortex, responsible for decision-making and impulse control, may become impaired. Understanding the neurobiology of addiction is crucial for the development of effective interventions and treatments. It informs the design of therapies that target specific brain regions and neurotransmitter systems to help individuals recover. By recognizing how alcohol rewires the brain's circuitry, healthcare professionals can tailor treatments to address these neurobiological changes, ultimately improving the outcomes of addiction treatment programs and helping individuals regain control over their lives [35].

Alcohol-Related Factors

Craving and withdrawal symptoms: Cravings for alcohol and the experience of withdrawal symptoms are significant challenges for individuals with alcohol dependence. Cravings can be intense and persistent, often triggered by cues associated with past alcohol use, stress, or social situations. These cravings can become even more pronounced after periods of abstinence, making it difficult for individuals to resist the urge to drink. Withdrawal symptoms, which can include anxiety, tremors, sweating, nausea, and even seizures in severe cases, can be physically and psychologically distressing. These symptoms typically arise when alcohol is no longer present in the body, and they can be powerful drivers of relapse as individuals may seek to alleviate their discomfort by drinking. Managing cravings and withdrawal symptoms is a critical component of alcohol addiction treatment, often addressed through medical and therapeutic interventions to help individuals cope with and overcome these challenges [36].

Triggers and temptations: Specific triggers and situations can create strong temptations for individuals in recovery from alcohol addiction. These triggers can include being in environments associated with past alcohol use, socializing with friends or acquaintances who still consume alcohol, or experiencing high-stress situations. These temptations can be challenging to resist, as they may be linked to pleasurable memories or perceived coping mechanisms. Identifying and avoiding these triggers is crucial for relapse prevention. This process often involves developing effective coping strategies and learning to navigate high-risk situations successfully. Cognitive-behavioral therapy and relapse prevention programs can help individuals recognize these triggers and build the skills needed to manage and reduce their impact, ultimately supporting long-term sobriety. Additionally, establishing a solid support network and making lifestyle changes that minimize exposure to triggering situations can be invaluable in maintaining recovery [36].

Assessment and prediction of alcohol relapse

Screening Tools and Assessment Methods

Clinical interviews: Clinical interviews are a fundamental component of assessing alcohol use and the risk of relapse. Healthcare providers conduct structured interviews with patients to gather information about their alcohol use history, patterns of consumption, triggers for drinking, and cravings. These interviews often include open-ended questions that encourage patients to share their experiences and standardized assessment tools to evaluate the risk of relapse. The information gathered during these interviews helps providers understand the patient's unique circumstances and tailor treatment and relapse prevention strategies accordingly [37].

Substance abuse questionnaires: Various validated substance abuse questionnaires, such as the Alcohol Use Disorders Identification Test (AUDIT) and the Michigan Alcohol Screening Test (MAST), are valuable tools in assessing alcohol misuse and determining the severity of the problem. These questionnaires consist of a series of questions that address different aspects of alcohol use, including frequency, quantity, and associated consequences. The patient's responses are scored to provide an overall assessment of their alcohol use and the risk of relapse. These tools offer a standardized and quantifiable way to evaluate the patient's alcohol use patterns [38].

Biomarkers: Monitoring specific biomarkers can provide objective evidence of recent alcohol use, making them a valuable component of relapse risk assessment. Biomarkers like carbohydrate-deficient transferrin (CDT) and ethyl glucuronide (EtG) can be detected in blood or urine samples. CDT, for instance, reflects heavy and chronic alcohol consumption, while EtG is a reliable marker of recent alcohol exposure. These biomarkers offer an objective and quantifiable way to confirm alcohol use, especially when patients may be reluctant to disclose their consumption during interviews or questionnaires [39].

Psychological assessments: Psychological assessments, including the evaluation of mental health and psychosocial factors, play a crucial role in identifying patients at risk of relapse. Underlying mood disorders, stress-related factors, or co-occurring psychiatric conditions can significantly increase the risk of relapse. Comprehensive psychological assessments can help uncover these factors and guide the development of treatment plans that address not only alcohol dependence but also the patient's mental and emotional well-being. These assessments may include standardized tests, interviews, and observations by mental health professionals [40].

Identifying High-Risk Patients

Past history of alcohol use: Patients with a significant history of alcohol abuse or a previous diagnosis of alcohol dependence are at a higher risk for relapse. The chronic and relapsing nature of alcohol use disorder can make individuals who have a history of heavy drinking more vulnerable to relapse. In the context of a liver transplant, it is essential to assess the patient's alcohol use history, patterns, and the circumstances surrounding their liver disease. Understanding their past experiences with alcohol is crucial for relapse prevention and ongoing support [36].

Lack of social support: Patients with limited or weak social support systems are more vulnerable to relapse. The presence of a supportive family and friends can be a protective factor in maintaining sobriety. Social support plays a significant role in the recovery process, as it provides emotional encouragement, accountability, and a safety net in times of temptation or crisis. Patients who lack a robust support network may struggle more when facing recovery challenges. As such, addressing social support and building a reliable post-transplant support system is a critical aspect of relapse prevention [41].

Co-occurring mental health disorders: Patients with comorbid mental health conditions, such as depression or anxiety, are at an increased risk of relapse. These mental health conditions can be both a cause and a consequence of alcohol abuse, as individuals may turn to alcohol as a coping mechanism for emotional distress. Identifying and addressing these co-occurring disorders is crucial for relapse prevention. Comprehensive mental health assessments and appropriate treatment, such as therapy and medication, can help individuals manage their mental health symptoms and reduce the risk of turning to alcohol for relief [33].

Previous relapses: Patients who have experienced relapses in the past, either before or after transplantation, are at a greater risk for subsequent relapse. Previous relapses are strong indicators of the challenges individuals face in maintaining sobriety. Understanding the circumstances and triggers of these relapses is essential for tailoring relapse prevention strategies. It's also important to emphasize that relapse is a standard part of the recovery process for some individuals, and it doesn't signify failure. Instead, it highlights the need for ongoing support and adjustments to the treatment plan [42].

Predictive Factors for Relapse

Cravings and temptations: The intensity and frequency of alcohol cravings, along with the presence of strong temptations to drink, are powerful predictors of relapse. Cravings can be triggered by various factors, including cues associated with past drinking, stress, and emotional states. When individuals experience intense and persistent cravings, it can become increasingly challenging to resist the urge to drink. Practical strategies for managing and coping with cravings are essential in relapse prevention [43].

Stress levels: High levels of stress, especially when coupled with poor stress-coping mechanisms, can significantly increase the risk of relapse. Stress is a common trigger for alcohol use, as individuals may turn to alcohol as a way to self-medicate and temporarily relieve stress and tension. When facing chronic or acute stressors, those in recovery may find it difficult to maintain sobriety. Effective stress management techniques and coping strategies are crucial in reducing this risk [44].

Socioeconomic factors: Socioeconomic factors, such as economic instability, unemployment, and housing issues, can contribute to the risk of relapse. These stressors may not only create emotional distress but also trigger alcohol use as a coping mechanism. Economic challenges can limit access to support services, therapy, and treatment, making it more difficult to maintain recovery. Addressing socioeconomic factors and providing resources for financial stability and housing support can be essential in reducing relapse risk [45].

Presence of triggers: Specific environmental or social triggers that remind patients of their previous drinking habits can significantly increase the risk of relapse. These triggers can be diverse, including places, people, or situations associated with past alcohol use. Patients must recognize and develop strategies for avoiding or effectively managing these triggers. This may involve making lifestyle changes, seeking alternative social activities, or practicing coping techniques to navigate triggering situations without alcohol [46].

Early abstinence period: The risk of relapse is often highest during the early stages of abstinence. Patients who have recently stopped drinking may face intense withdrawal symptoms, cravings, and the challenge of adapting to a sober lifestyle. However, it is important to note that the risk of relapse tends to decrease over time. Patients who successfully maintain sobriety for a more extended period often build stronger recovery foundations and generally have a lower risk of relapse. Continuous support and relapse prevention strategies are critical, particularly during the early stages of abstinence [42].

Strategies for preventing alcohol relapse

Pre-transplant Interventions

Alcohol abstinence programs are a crucial component of the pre-transplant preparation for individuals seeking organ transplantation. These programs play a vital role in assisting patients in achieving and sustaining sobriety before they become eligible for transplantation. Such programs typically encompass a range of interventions, including structured counseling, educational sessions on the potential risks of alcohol relapse, and personalized approaches tailored to meet the specific needs of each patient. These initiatives are designed not only to ensure that the patients abstain from alcohol but also to help them understand the importance of sobriety in the context of a successful transplant. By engaging patients in these programs, medical professionals can work toward creating a foundation of sobriety that enhances the overall success of transplantation procedures [47].

In addition to alcohol abstinence programs, psychosocial support is an equally critical aspect of the pre-transplant process for individuals grappling with alcohol addiction. These support programs are designed to address the emotional and psychological aspects of addiction, recognizing that overcoming alcoholism is not just about physical abstinence but also about managing the complex psychological and emotional challenges that addiction can present. Psychosocial support includes various therapeutic and counseling approaches aimed at equipping patients with the tools and strategies needed to cope with emotional stressors, triggers, and the mental hurdles associated with addiction. By providing this support, healthcare professionals ensure that patients are not only physically ready for the transplant but also mentally prepared, thus increasing their chances of a successful and sustainable recovery post-transplant. This holistic approach, which combines alcohol abstinence programs and psychosocial support, contributes to the overall well-being and recovery of patients undergoing organ transplantation while addressing the multifaceted nature of addiction [48].

Post-transplant Interventions

Alcohol monitoring and testing play a pivotal role in the post-transplant phase, contributing significantly to relapse prevention. Regular and systematic alcohol monitoring and testing serve as a safety net for patients who have undergone transplantation, as they help in the early detection of any relapse or return to alcohol use. These tests can be administered randomly or on a scheduled basis, ensuring that no lapse in sobriety goes unnoticed. When relapse is identified in its early stages, healthcare providers can intervene promptly, offering timely support, counseling, and interventions to prevent the re-establishment of problematic alcohol consumption patterns. In this way, alcohol monitoring and testing serve as a critical safeguard, promoting the long-term success of the transplant and the patient's overall well-being [49].

Cognitive-behavioral therapy (CBT) is another vital component of addiction treatment for post-transplant patients. CBT is a widely recognized therapeutic approach that focuses on identifying and modifying the thought patterns and behaviors associated with alcohol use. By addressing the underlying causes and triggers of alcohol addiction, CBT empowers patients to develop healthier coping strategies and more adaptive responses to stressors, cravings, and other factors that may lead to relapse. This form of therapy equips patients with the necessary skills and tools to not only achieve initial abstinence but also to sustain sobriety in the long term, making it an essential part of the recovery process [50].

Medication-assisted therapy offers another valuable option in preventing relapse for transplant recipients with a history of alcohol addiction. Medications like naltrexone and acamprosate are used to reduce cravings and decrease the appeal of alcohol by acting on the brain's reward system. These medications help to mitigate the physical and psychological components of alcohol addiction, making it easier for patients to resist the temptation of alcohol, even in the face of triggers and stressors. Medication-assisted therapy can be particularly effective in combination with other therapeutic approaches, enhancing the overall success of relapse prevention strategies and promoting lasting recovery [51].

Multidisciplinary Care and Support Teams

Transplant team collaboration is a fundamental aspect of providing comprehensive care to individuals who have undergone transplantation and are in recovery from alcohol addiction. These multidisciplinary care teams, composed of transplant coordinators, hepatologists, addiction specialists, psychologists, and social workers, work together to ensure patients receive holistic support. The collaborative expertise of these professionals ensures that the patient's physical, psychological, and emotional needs are addressed in a coordinated manner. This approach not only enhances the quality of care but also supports the successful integration of the transplant into the patient's life, creating a strong foundation for sustained sobriety [52].

Ongoing psychosocial support is essential for the long-term recovery of post-transplant patients. Regular counseling sessions and participation in support groups provide patients with a continuous source of guidance and assistance in addressing the challenges they may face. These psychosocial support mechanisms are designed to help patients cope with the emotional and psychological aspects of addiction and recovery, thereby aiding in maintaining their sobriety over the long term [53].

Education and patient engagement are vital in empowering individuals to take control of their recovery. Educating patients about the risks associated with alcohol relapse and involving them in their care can be highly motivating. When patients have a clear understanding of the potential consequences of relapse, they are more likely to remain committed to their journey of abstinence. This collaborative approach fosters a sense of responsibility and self-determination in patients, enhancing their success in sustaining sobriety [54].

Family involvement is another critical element in the care process. Engaging family members in the patient's recovery journey is essential, as their support and involvement can significantly contribute to the patient's overall well-being and reduce the risk of relapse. The family's understanding of the patient's challenges, along with their encouragement and assistance, creates a more supportive environment for recovery [55].

Relapse response plans provide a safety net in case a patient does experience a relapse. Developing comprehensive response plans ensures that if a patient does relapse, there is a swift and effective response in place to address the situation. These plans may include treatment adjustments, increased counseling, and additional support measures. Having such plans in place can help minimize the potential harm associated with a relapse, facilitating a quicker return to sobriety and reducing the risk of repeated relapses [51].

Future directions and research

Emerging Trends in ALD Treatment and Transplantation

Personalized medicine: The concept of personalized medicine involves tailoring medical treatment to the specific characteristics of an individual patient. In the context of ALD, personalized medicine considers a patient's unique genetic and clinical features. This approach is becoming increasingly important in treating ALD because it recognizes that not all patients respond to treatments similarly. By analyzing an individual's genetic makeup and disease profile, healthcare providers can make more informed decisions about the most suitable treatment options. This may lead to more effective and targeted therapies, minimizing the risk of adverse effects and improving overall patient outcomes [56].

Novel therapies: Research continually explores new and innovative therapeutic approaches for ALD. Some of the most promising areas of study include stem cell therapy, gene therapy, and immunomodulatory treatments. Stem cell therapy involves using specialized cells to repair damaged liver tissue. Gene therapy seeks to correct the underlying genetic factors contributing to ALD. Immunomodulatory treatments aim to modulate the immune system's response to reduce inflammation and tissue damage. These emerging therapies could revolutionize ALD treatment by providing additional options beyond traditional approaches like immunosuppressants or liver transplantation [57].

Early detection: Early detection of ALD is crucial for successful disease management. Advances in diagnostic techniques and the identification of biomarkers make it easier to diagnose ALD at earlier stages. Early detection allows for prompt intervention, which is vital in preventing disease progression and minimizing liver damage. By catching ALD in its early phases, the need for more invasive and complex treatments, such as liver transplantation, can be reduced. This is a significant advancement as it improves the overall prognosis and quality of life for ALD patients [58].

Artificial liver devices: Research into artificial liver devices and bioartificial livers is another area of great importance for ALD patients. These technologies aim to provide temporary support for individuals with ALD while they are awaiting transplantation or recovering from acute liver injury. An artificial liver device can assist in performing some of the essential functions of the liver, such as filtering toxins from the blood and regulating various metabolic processes. This can be a critical bridge for patients awaiting a liver transplant, as it helps maintain their health until a suitable donor organ becomes available. Additionally, bioartificial livers, which incorporate living cells, may offer more advanced liver support by providing a more natural environment for liver function [59].

Research Gaps and Areas for Future Studies

Alcohol relapse predictors: Understanding the factors that predict alcohol relapse in ALD patients is a critical research area. Relapse in alcohol use can have detrimental effects on the already compromised liver, and identifying robust predictors is essential for designing effective interventions. These predictors can encompass various factors, including genetic markers, neurobiological changes, and psychosocial influences. By pinpointing the specific risk factors for relapse, healthcare providers can better tailor treatment and support for ALD patients, increasing the chances of sustained recovery [60].

Psychosocial interventions: ALD patients face unique psychosocial challenges, both before and after liver transplantation. Developing and evaluating psychosocial interventions that address these challenges is vital. These interventions may include counseling, support groups, and strategies to cope with the emotional and social aspects of living with ALD or undergoing transplantation. Adequate psychosocial support can improve the overall well-being and quality of life for ALD patients and contribute to their successful recovery [61].

Long-term outcomes: While many studies focus on short-term outcomes and immediate post-transplant success, there is a need for long-term studies that track the progress of ALD transplant recipients over extended periods. This research is essential to gain a more comprehensive understanding of the effectiveness of current interventions and the risk of relapse over time. Long-term studies can help healthcare providers and researchers identify trends and challenges that might take time to become apparent, leading to improved treatment strategies and more realistic expectations for patients and healthcare providers [62].

Organ shortage solutions: The shortage of donor organs, including livers, is a significant challenge in transplantation medicine. In the context of ALD, where transplantation is often a life-saving measure, addressing this shortage is paramount. Exploring innovative solutions, such as expanded criteria for donor selection, is one approach. This might involve using organs from donors who might not meet traditional criteria but could still provide a suitable match for certain patients. Additionally, the development of bioengineered livers or other artificial organ technologies can revolutionize transplantation by reducing the reliance on human donors and expanding the pool of available organs. These solutions can significantly impact the feasibility and success of liver transplantation for ALD patients [63].

Innovations in Relapse Prevention

Telemedicine and digital health: Telemedicine and digital health platforms can significantly improve post-transplant care for ALD patients. These technologies enable remote consultations with healthcare providers, making it easier for patients to access follow-up care and support, especially in cases where regular in-person visits may be challenging. Digital health tools can also provide continuous monitoring, counseling, and education, helping patients manage their condition effectively. These platforms can enhance accessibility, engagement, and communication between patients and healthcare teams, ultimately improving outcomes [64].

Pharmacogenomics: Advances in pharmacogenomics involve tailoring medication regimens based on an individual's genetic profile. For ALD patients, this can be especially valuable in medication-assisted therapy. By analyzing a patient's genetic makeup, healthcare providers can better predict which medications are most effective and well-tolerated, reducing the need for trial-and-error approaches. This personalized approach can improve medication management and outcomes while minimizing the risk of adverse reactions [65].

Wearable devices: Wearable devices that monitor vital signs and other relevant biomarkers can be integrated into relapse prevention strategies for ALD patients. These devices can track alcohol metabolites, monitor physical health, and provide real-time alerts to patients and healthcare providers. This continuous monitoring can help detect early warning signs of relapse and enable timely interventions. Wearable devices also encourage patients to take an active role in their recovery and make them more aware of their health status [66].

Artificial intelligence (AI) and predictive analytics: AI and predictive analytics can assess a patient's risk of relapse by analyzing medical, behavioral, and psychosocial data. AI algorithms can identify patterns and trends that might not be apparent to human observers, allowing for early intervention and support. By providing timely reminders, interventions, and personalized recommendations, AI can help individuals with ALD maintain their sobriety and reduce the risk of relapse [67].

Supportive mobile applications: Mobile applications explicitly designed for ALD patients can offer various resources and support. These apps can provide educational materials, connect patients to support communities, offer crisis helplines, and track personal progress. They are a convenient and accessible tool for patients to access information and assistance, especially during critical moments when they need support. Such applications can enhance patient engagement and provide a sense of community and ongoing care [68].

## Conclusions

In conclusion, the comprehensive review of alcohol relapse in liver transplant patients with ALD underscores the complexity and critical nature of this issue. ALD represents a significant public health concern, with liver transplantation offering a lifeline to those in the advanced stages of the disease. However, the persistent risk of alcohol relapse post-transplantation remains a formidable challenge. This review has highlighted key findings and insights, emphasizing the importance of rigorous pre-transplant assessments, effective post-transplant interventions, and multidisciplinary care teams in mitigating this risk. Furthermore, it emphasizes the need for a holistic approach to ALD and transplantation that considers not only the medical aspects but also the psychosocial and psychological well-being of patients. With such an approach, we can work toward better patient outcomes, reduced relapse rates, and improved quality of life for individuals battling ALD.
